# Tunable Electrical Conductivity and Simultaneously Enhanced Thermoelectric and Mechanical Properties in n‐type Bi_2_Te_3_

**DOI:** 10.1002/advs.202203250

**Published:** 2022-07-28

**Authors:** Lu‐Yao Lou, Jianmin Yang, Yu‐Ke Zhu, Hao Liang, Yi‐Xin Zhang, Jing Feng, Jiaqing He, Zhen‐Hua Ge, Li‐Dong Zhao

**Affiliations:** ^1^ Faculty of Materials Science and Engineering, Kunming University of Science and Technology Kunming Kunming 650093 China; ^2^ Shenzhen Key Laboratory of Thermoelectric Materials and Department of Physics Southern University of Science and Technology Shenzhen 518055 China; ^3^ School of Materials Science and Engineering Beihang University Beijing 100191 China

**Keywords:** Bi_2_Te_3_, Na doping, power factor, thermoelectrics, thermal conductivity

## Abstract

The recent growing energy crisis draws considerable attention to high‐performance thermoelectric materials. n‐type bismuth telluride is still irreplaceable at near room temperature for commercial application, and therefore, is worthy of further investigation. In this work, nanostructured Bi_2_Te_3_ polycrystalline materials with highly enhanced thermoelectric properties are obtained by alkali metal Na solid solution. Na is chosen as the cation site dopant for n‐type polycrystalline Bi_2_Te_3_. Na enters the Bi site, introducing holes in the Bi_2_Te_3_ matrix and rendering the electrical conductivity tunable from 300 to 1800 Scm^–1^. The solid solution limit of Na in Bi_2_Te_3_ exceeds 0.3 wt%. Owing to the effective solid solution, the Fermi level of Bi_2_Te_3_ is properly regulated, leading to an improved Seebeck coefficient. In addition, the scattering of both charge carriers and phonons is modulated, which ensured a high‐power factor and low lattice thermal conductivity. Benefitting from the synergistic optimization of both electrical and thermal transport properties, a maximum figure of merit (*ZT)* of 1.03 is achieved at 303 K when the doping content is 0.25 wt%, which is 70% higher than that of the pristine sample. This work disclosed an effective strategy for enhancing the performance of n‐type bismuth telluride‐based alloy materials.

## Graphical abstract

In this study, Na as the n‐type dopant is introduced in the polycrystalline Bi_2_Te_3_ material for enhancing the thermoelectric and mechanical properties. Benefitting from the synergistic optimization of electrical and thermal transport properties, a maximum *ZT* value of 1.03 is achieved at 303 K for the Bi_2_Te_3_‐0.25 wt% sample, which is 70% higher than that of the pristine sample.

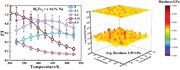

## 1 Introduction

The current low efficiency of energy utilization not only causes energy waste but also indirectly contributes to global climate issues.^[^ ^1^ ^]^ Thermoelectric (TE) materials can realize the direct conversion between thermal energy and electrical energy in an environmentally friendly way, thus causing extensive concern in recent years.^[^ ^2^, ^3^, ^4^ ^]^ Thermoelectric properties can be evaluated by the dimensionless figure of merit (*ZT)*, which is defined as *ZT* = *S* ^2^ *σT*/*κ*, where *S* is the Seebeck coefficient, *σ* is the electrical conductivity, *κ* is the total thermal conductivity, and *T* is the absolute temperature.^[^ ^5^ ^]^ From this equation, the high *ZT* value requires the coexistence of high electrical transport properties (*S* and *σ*) and low thermal conductivity (*κ*). However, it is difficult to decouple *S*, *σ*, and *κ* because of the inner relationship among them.^[^ ^6^, ^7^ ^]^

In the past two decades, rapid progress has been achieved for thermoelectric materials due to the exploration As the only commercial thermoelectric material with high near‐room‐temperature performance and excellent processability, bismuth telluride was intensively investigated during this period.^[^ ^8^, ^9^, ^10^ ^]^ Due to the target of practical applications, the majority of research has focused on polycrystal bismuth telluride based on powder metallurgy.^[^ ^9^ ^]^ The TE performance of polycrystal Bi_2_Te_3_ was thereby enhanced by many optimization strategies, such as energy filtering,^[^ ^11^, ^12^, ^13^ ^]^ band engineering,^[^ ^14^, ^15^, ^16^ ^]^ texturing,^[^ ^8^, ^17^, ^18^, ^19^ ^]^ and defect engineering.^[^ ^10^, ^20^, ^21^, ^22^ ^]^ In particular, an outstanding *ZT* value of 1.86 was achieved in a p‐type Bi_2_Te_3_ alloy via a liquid phase sintering strategy.^[^ ^20^ ^]^

In contrast, although it has also been attempted multiple times, the n‐type Bi_2_Te_3_ still behaves with much lower TE performance, particularly at near room temperature. One of the reasons for this outcome is that the weakened anisotropy by the polycrystalline process is more harmful to n‐type Bi_2_Te_3_, which can be resolved by texturing to some extent.^[^ ^8^ ^]^ However, because the repeatedly hot‐forging process for texturing is time‐consuming and strenuous,^[^ ^17^, ^18^, ^19^ ^]^ other reasons for the inferior TE performance still deserve attention. It was reported that mechanical deformation can intensively produce a donor‐like effect in Bi_2_Te_3_.^[^ ^21^, ^23^, ^24^, ^25^ ^]^ The significantly increased n‐type charge carriers would thereby deviate the carrier concentration to the optimum value.^[^ ^26^ ^]^ It is reasonable that reducing the carrier concentration would be available for powder‐processed, n‐type Bi_2_Te_3_, which is generally realized by alloying or doping.^[^ ^9^, ^10^ ^]^ For n‐type Bi_2_Te_3_ that is traditionally alloyed with Se to synergistically optimize the point defects and band structure, Pan et al. further regulated the Se content in powder‐processed (Bi, Se)_2_Te_3_ to reduce the carrier concentration.^[^ ^26^ ^]^ However, although the peak *ZT* value was increased, the increased Se also enlarged the bandgap that moved the peak *ZT* to a high temperature. In addition, doping with the element of +1 or +2 valance in the cation site was theoretically effective in reducing the carrier concentration. However, it is conventionally considered that doping at the cation site can easily affect the conduction band structure,^[^ ^27^ ^]^ which is adverse to maintaining the high transport properties. Therefore, only a few reports about n‐type Bi_2_Te_3_ have focused on cation site doping. However, this strategy is still worthy of further attention.

Considering that cation site doping may affect the band structure,^[^ ^27^ ^]^ pristine Bi_2_Te_3_ without band engineering by Se was selected as the research object. The material exhibited strong n‐type properties due to the donor‐like effect introduced by high‐energy ball milling. Sodium doping was subsequently conducted on this n‐type Bi_2_Te_3_ because of its +1 valance in the cation site. In addition, the similar ionic radius of Na^+^ (Na^+^: 102 pm) to Bi^3+^ (Na^+^: 102 pm, Bi^3+^: 103 pm) would make it more stable in the cation site, unlike Cu^+^ (73 pm), which would move to the Van de Waals gap of the Bi_2_Te_3_ matrix.^[^ ^28^, ^29^ ^]^ The results show that a small amount of Na may not affect the band structure but can effectively reduce the carrier concentration. The modified defects can also synergistically optimize both charge carrier and phonon transport. The Na content of 0.25 wt% was determined to significantly increase the peak *ZT* value to 1.03 and move it closer to room temperature. The result of this work affords a significant guide for cation site doping in n‐type bismuth telluride.

## 2 Results and Discussion

**Figure 1:**
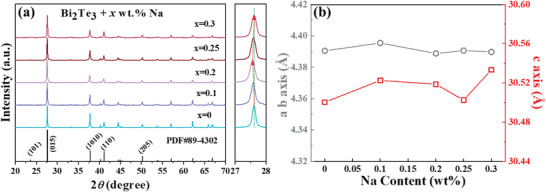
a) Powder XRD patterns and b) refined lattice parameters for Bi_2_Te_3_ with different Na contents.

The X‐ray diffraction patterns for the crushed powders of all samples are plotted in **Figure** **1**a. All samples matched the standard PDF card (PDF#89‐4302). No peaks for the impurity were detected even after doping with Na. The right side of Figure 1a demonstrates the magnified view of the (015) peaks. Note that the peak gradually shifted to a lower angle with an increase in Na content for *x* ≤ 0.2 and then shifted to a higher angle when the Na content further increased. These shifting peaks indicate that the lattice first expanded and then shrank with an increase in Na content, which can be understood by the behavior of the doped Na. The cation substitution of Bi^3+^ by Na^+^ could be expressed by:

(1) $$xNa+Bi_{2}Te_{3}\to xN{a^{\prime \prime }}_{Bi}+\left(2-x\right)Bi_{Bi}+3Te_{Te}+2xh^{\cdot }$$

This reaction is the main reason of the donor concentration reduction. The introduction of Na is equivalent to making the sample Te‐deficient. Under this condition, anion vacancies $V_{\mathrm{Te}}^{\cdot \cdot \cdot }$ can be generated and rapidly occupied by Bi to form antisite defects Bi′_Te_, thereby inflating the lattice, which happened in Zn doping cation sites as well.^[^ ^30^ ^]^ These changes can be more precisely reflected by the refined lattice parameter, as shown in Figure 1b, which also reveals more details about the generated Bi′_Te_. Lattice parameter *c* exhibited obvious expansion compared with the lattice parameters *a* and *b*.^[^ ^31^ ^]^ It is well known that Bi_2_Te_3_ consists of the quintuple‐layer unit ‐Te(1)‐Bi‐Te(2)‐Bi‐Te(1)‐, which interconnects by van der Waals forces. As explored by Hashibon et al.,^[^ ^32^ ^]^ the antisite defects Bi′_Te_ in Bi_2_Te_3_ are more inclined to be generated at Te(1) sites. Therefore, the increased Bi′_Te_ at the Te(1) site with added Na may enlarge the van der Waals gap between the two Te(1) layers. When the Na content increases to a certain degree (*x* = 0.25), the formation energy of $V_{\mathrm{Te}}^{\cdot \cdot \cdot }$ becomes lower than that of Bi′_Te_ due to the excessive Te deficiency. The generated $V_{\mathrm{Te}}^{\cdot \cdot \cdot }$ then shrank the lattice and decreased the density.^[^ ^9^ ^]^

**Figure 2:**
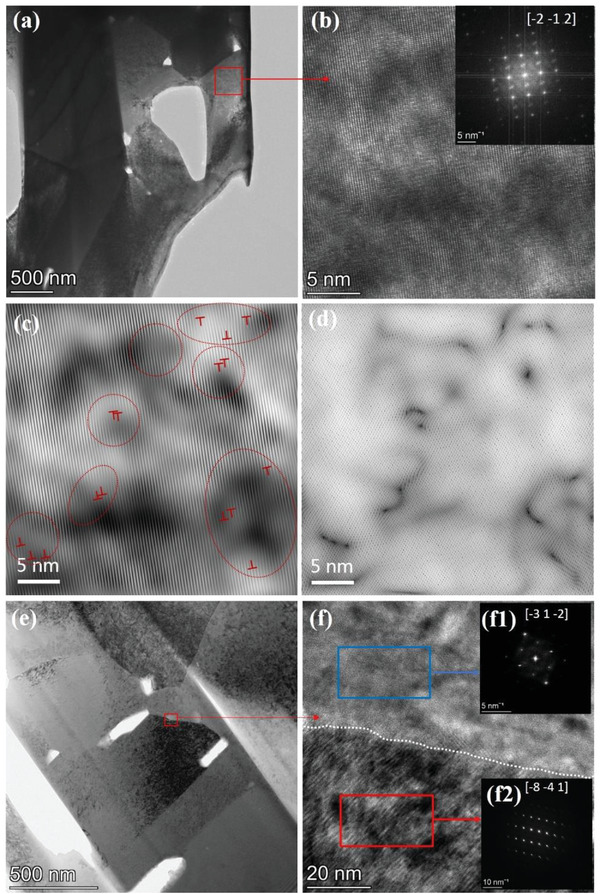
a) Low magnification TEM image for Bi_2_Te_3_+0.25 wt% Na sample. b) High‐resolution TEM image corresponding to the red rectangle marked in (a), with the inset of electron diffraction pattern. c,d) the inverse Fourier transform images of the sample obtained by (b), which reveal the dislocation distribution (marked by red cycles) and the accompanied lattice distortion around the dislocations. f) High‐resolution TEM image of the phase interface area, corresponding to the red rectangle marked in e), grain boundary (marked with a white dotted line) with two corresponding electron diffraction patterns given in (f1) and (f2), which correspond to the blue and red rectangles marked in (f), respectively. The segregation is Na_2_TeO_4_ in the direction of [‐3 1‐2].

**Figure** **2**a shows the low‐magnification transmission electron microscope (TEM) images of the Bi_2_Te_3_+0.25 wt% Na sample, and strip‐like grains and pores are observed. Figure 2b shows the high‐resolution, TEM image corresponding to the red rectangle marked in Figure 2a, with a corresponding fast fourier transform (FFT) image given in the inset, which shows agreement with the crystal direction [‐2‐1 2] of Bi_2_Te_3_. The inverse Fourier transform images obtained in Figure 2b are displayed in Figure 2c,d, which reveal the dislocation distribution and the accompanied lattice distortion around the dislocations. The existence of dislocations is in favor of scattering mid‐frequency phonons to deteriorate the lattice thermal conductivity as well as strengthening the mechanical performance of materials. Figure 2f shows the high‐resolution, TEM image corresponding to the red rectangle marked in Figure 2e, showing a grain boundary (marked with a white‐dotted line). Two corresponding FFT images are given in Figure 2f, 1,f2), which correspond to the blue and red rectangles marked in Figure 2f, respectively, indicating that the precipitate is Na_2_TeO_4_ with the direction of [‐3 1‐2]. This result shows agreement with the EDS mapping shown in Figure S1, Supporting Information. The Na_2_TeO_4_ nanoprecipitates may be beneficial to the low lattice thermal conductivity of Bi_2_Te_3_ bulk materials.

The fractured surfaces of samples with different Na contents were characterized by scanning electron microscope (SEM) and are shown in Figure S2, Supporting Information. All samples exhibited intergranular fracture with good crystallinity. The grains showed a lamellar morphology with an average size of ≈5 µm. These results indicate that adding Na would not deteriorate the quality of the sintering process. In addition, the grains were all randomly oriented for samples with different Na contents, which suggested that adding Na was not assisted in the orientated alignment of grains. All samples contain some micropores at the grain boundaries, while no obvious changes in the number of micropores were observed. However, the density of the sample gradually increased with an increase in Na content, as shown in Figure S2f, Supporting Information. The increased density might be attributed to the increased antisite defect amount, which was consistent with the theoretical density calculated by Miller et al. from the antisite point model.^[^ ^9^, ^33^ ^]^

**Figure 3:**
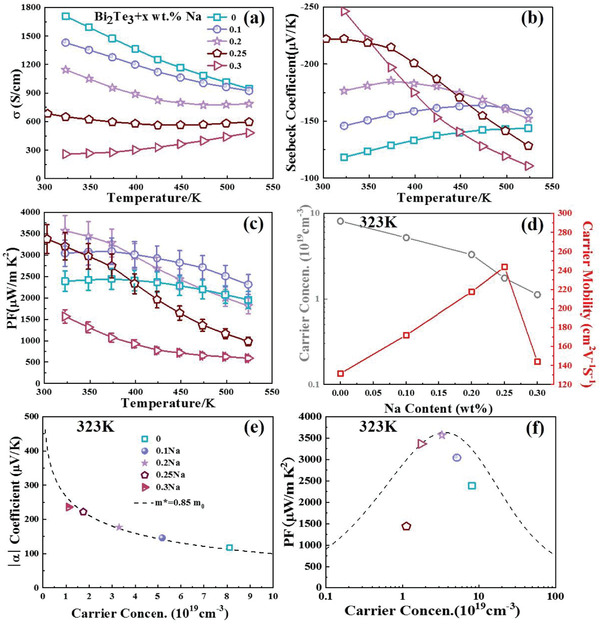
Temperature dependence of a) electrical conductivity, b) Seebeck coefficient, c) power factor for Bi_2_Te_3_ with different Na contents; d) carrier concentration and mobility at 323 K for Bi_2_Te_3_ with different Na contents; carrier concentration dependence of e) absolute value of the Seebeck coefficient and f) power factor at 323 K.

**Figure** **3** shows the temperature‐dependent, electrical transport properties for the samples with different Na contents. The specific values of the electrical transport properties at 323 K are exhibited in **Table** **1**. The *σ* generally decreased with an increase in Na content, as shown in Figure 3a. The *σ* value was 1710 S cm^–1^ at 323 K for the sample without Na. When the Na content increased to *x* = 0.25, the *σ* value decreased to 683 S cm^–1^ at 323 K. The Na content monotonically decreased with an increase in temperature when *x* ≤ 0.2, indicating the property of degenerate semiconductors. However, the decline gradually slowed with an increase in Na content and even increased at high temperatures. The Seebeck coefficient (*S*) overall increased with an increase in Na content, especially in the low‐temperature range, as shown in Figure 3b. The *S*‐value at 323 K increased ≈88% from 118 µV K^–1^ to 222 µV K^–1^ when *x* increased from 0 to 0.25, the optimized carrier concentration is the main reason.^[^ ^34^ ^]^ The results showed a trend of first increasing and then decreasing for *x* ≤ 0.2, of which the peak gradually shifted to a lower temperature with an increase in Na content. When *x* > 0.2, the peak shifted out of the measuring temperature range and caused *S* to show a monotonically decreasing trend. The variation in bandgap (*E*_g_) can also be reflected by the shifted *S* peaks according to the Goldsmid–Sharp band gap estimation:^[^ ^35^ ^]^

(2) $$E_{g}=2e{\left|S\right|}_{\max}T_{\max}$$

where |*S*|_max_ is the absolute value of the maximum *S* and T_max_ is the temperature at which |*S*|_max_ is observed. The calculated *E*_g_ values are shown in Table 1. Note that *E*_g_ gradually shrank with an increase in Na content. The reason for the decreased *S* with an increase in temperature was the intrinsic excitation of narrow bandgap semiconductors, which have been widely reported in multiple studies.^[^ ^9^, ^36^ ^]^ The more easily occurring intrinsic excitation caused by the narrowed band gap moved the *S* peak forward, which also explains the change in *σ* against the Na content. The change in *σ* and *S* correspondingly contributes to the variation in the power factor (PF), as shown in Figure 3c. The PF overall increased when *x* ≤ 0.2. When *x* = 0.25, the PF still demonstrates a significant increase at temperatures lower than 398 K, while it considerably decreases in the high‐temperature range. A high PF value of 3377 µW m^−1^ K^−2^ was obtained at 303 K for the *x* = 0.25 sample. A further increase in the Na amount significantly decreased the PF. Therefore, the proper regulation of Na content can optimize the electrical transport properties.

**Table 1:** Electrical transport properties, bandgap, and transverse sound velocity at 323 K for Bi_2_Te_3_ with different Na contents

*x*	*σ* [S cm^–1^]	*S* [µV K^–1^]	*n*_H_ [10^19^ cm^–3^]	*µ*_H_ [cm^2^ V^–1^ S^–1^]	*E*_g_ [eV]	*v*_T_ [m s^–1^]
0	1710	−118	8.11	131.60	0.241	1761
0.1	1430	−146	5.19	171.97	0.235	1751
0.2	1150	−177	3.3	217.50	0.222	1710
0.25	683	−222	1.75	243.60	0.215	1693
0.3	258	−246	1.12	143.77	‐	1678

The intrinsic reasons for the variation in the electric transport properties can be clarified by the Hall carrier concentration (*n*_H_) and Hall mobility (*µ*_H_) shown in Figure 3d. Note that *n*_H_ decreased with an increase in Na content. As known by the single parabolic band (SPB) model, *S* can be expressed as follows^[^ ^3^, ^37^ ^]^:

(3) $$\begin{array}{c} S=-\frac{K_{B}}{e}\left[\frac{\left(S+\frac{5}{2}\right)F_{S+\frac{3}{2}}\left(\eta \right)}{S+\frac{3}{2}F_{S+\frac{1}{2}}\left(\eta \right)}-\eta \right]=-\frac{2K_{B}^{2}{\mathrm{Tm}}_{d}^{*}}{3e\hbar^{2}}{\left(\frac{\pi }{3n}\right)}^{\frac{2}{3}}\left(\frac{3}{2}+S\right) \end{array}$$

where *k*_B_ is Boltzmann's constant, *ħ* is the reduced Planck constant, $m_{d}^{*}$ is the density‐of‐state (DOS) effective mass, *n* is the carrier concentration, *η* = *E*_F_ */*(*k*_B_T) is the reduced Fermi level, and *s* is the scattering factor, which is −1/2 for acoustic‐phonon scattering. *F*_j_(*η*) is the Fermi‐Dirac distribution function, which can be expressed as follows:

(4) $$F_{j}\left(\eta \right)=\int_{0}^{\infty }\frac{\varepsilon^{j}d\varepsilon }{1+e^{\varepsilon -\eta }}$$

where *ε* = *E/*(*k*_B_T) is the reduced energy. According to Equations (1) and (2), the Pisarenko curve at 323 K is plotted and shown in Figure 3e, which illustrates that the effective mass ($m_{d}^{*}$) matches 0.85 *m*_0_ when *x* ≤ 0.25. The decreased carrier concentration via the movement of *E*_F_ would be the main reason for the increased *S*. Although this decrease would also cause a decrease in *σ*, the increased carrier mobility would compensate for the decrement. The *µ*_H_ increased with an increase in Na content when *x* ≤ 0.25, which may be attributed to the reduced interactional scattering by charge carriers. The sharply decreased *µ*_H_ when *x* = 0.3 may be attributed to the excessively charged point defect that changed the scattering mechanism or perhaps the change band structured by Na doping. This speculation can be supported by the large deviation in the point to the fitting curve for the *x* = 0.3 sample.

Based on the fitted $m_{d}^{*}$ of 0.85 *m*_0_, the theoretical PF can also be predicted by the single parabolic band (SPB) model. In the case of acoustic‐phonon scattering, the relation between carrier mobility and the nondegenerate limits of drift mobility (*µ*_0_) can be described as:

(5) $$u=u_{0}\frac{F_{-\frac{1}{2}}\left(\eta \right)}{2F_{0}\left(\eta \right)}$$

Therefore, according to Equation (1) and *σ* = *neµ*, the curve for the carrier concentration dependence of the theoretical PF at 303 K is plotted in Figure 3f with the data points of the experimental values. Note that the points were shifted closer to the peak of the curve when *x* increased to 0.2. Further increasing *x* would move the points far from the peak. This result more clearly illustrated the role of Na doping in optimizing the carrier concentration. In addition, the point for the *x* = 0 sample is far below the curve, showing a much lower *µ*_0_. The increased *µ*_0_ when increasing *x* to 0.2 indicates that the microstructure and the defect composition of the sample became more favorable for charge carrier transport.

**Figure 4:**
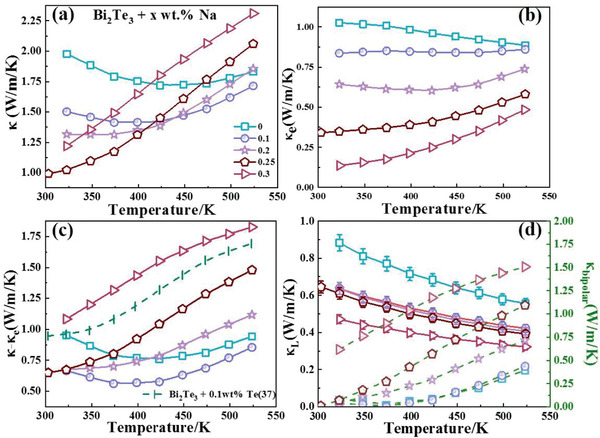
The temperature dependence of a) total thermal conductivity, b) electronic thermal conductivity, c) difference between the total thermal conductivity and the electronic thermal conductivity, and d) lattice thermal conductivity and bipolar thermal conductivity for Bi_2_Te_3_ with different Na contents.

In addition to the improved electrical transport properties, the thermal transport properties were also optimized by Na doping, as shown in **Figure** **4**. *κ* generally decreased with an increase in Na content (≤0.25 wt%), especially near room temperature. *κ* was 1.97 W m^−1^ K^−1^ for the undoped sample at 323 K. After adding 0.25 wt% Na, this value decreased by nearly half to 1.02 W m^−1^ K^−1^. The decrease in *κ* was contributed by both electronic transport and phonon transport. As shown in Figure 4b, because the electronic thermal conductivity (*κ*_e_) is strongly correlated with *σ*, it also decreased with an increase in Na content. As previously analyzed, the main reason should be the decreased carrier concentration. However, the decreased carrier concentration and narrowed *E*_g_ also produced an enhanced bipolar effect when the temperature increased. As a result, the more Na that was added, the higher the bipolar thermal conductivity (*κ*_bipolar_) at high temperatures (Figure 4d). The *x* = 0.3 sample even shows a significant *κ*_bipolar_ at 323 K, which differed from the other four samples. Therefore, the bandgap of the *x* = 0.3 sample should be reduced. The changed band structure conformed to the result of the Pisarenko curve shown in Figure 3e. The enhanced bipolar effect thereby caused an increase in *κ* at high temperatures, which was detrimental to the TE performance.

The lattice thermal conductivity (*κ*_L_) also significantly decreased with an increase in Na content at the whole measuring temperature, as shown in Figure 4d. Even the sample with a small amount of Na would show a sudden decrease in *κ*_L_. As known by the scattering mechanism of phonons,^[^ ^38^ ^]^ phonon scattering by point defects occurs as a relaxation time of *τ*_PD_ ≈ *ω* ^−4^, which focuses on high‐frequency phonons. Therefore, the reduced *κ*_L_ contributed by the point defect scattering of phonons should be more significant in the high‐temperature range. The decrease in the whole temperature range indicated other reasons that must explain these findings. As exhibited in Table 1, the transverse sound velocity (*v*_T_) of the samples monotonically decreased with an increase in *x*, indicating that the bond stiffness might be weakened by Na doping. This lattice softening might explain the reduced *κ*_L_. However, note that the decrement of *v*_T_ did not match *κ*_L_ and was a reasonable inference that *κ*_L_ might be intensively scattered by other effects focused on low‐ or mid‐frequency.

The representative scattering effect on low‐ or mid‐frequency phonons was caused by grain boundary (*τ*_B_ ≈ *ω* ^0^) and dislocation (*τ*_DC_ ≈ *ω* ^–3^ for dislocation cores and *τ*_DS_ ≈ *ω* ^–1^ for dislocation strain). Nevertheless, the similar grain size shown in Figure 2 indicates that grain boundary scattering was not a factor. Therefore, the introduction of dislocation, which reduced *κ*_L_ near room temperature, might be a factor. The point defects introduced by Na doping were quite helpful for the formation of line defects such as dislocations. Many studies have reported that dislocations can significantly reduce the *κ*_L_ of Bi_2_Te_3_‐based materials near room temperature.^[^ ^20^, ^22^, ^39^ ^]^ The internal strain induced by the dislocations could also have an important role in decreasing sound velocity (refer to Table 1).^[^ ^22^ ^]^ Therefore, it is highly possible that phonon scattering and lattice softening by dislocations significantly reduce *κ*_L_ after adding Na. Despite the increased *κ*_bipolar_, the *κ – κ*_L_ substantially decreased due to the reduced *κ*_L_, especially at near room temperature, as shown in Figure 4e. This value was lower than the reported values decreased by adding excessive Te (green curve in Figure 4e).^[^ ^40^ ^]^

**Figure 5:**
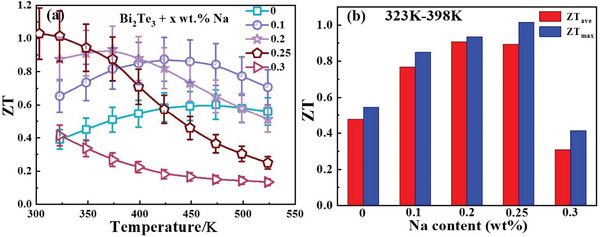
Temperature dependence of the *ZT* value for Bi_2_Te_3_ with different Na contents.

Benefitting from the synergistic optimization of the electrical and thermal transport properties, the total TE performance was significantly improved. As shown in **Figure** **5**, the peak *ZT* gradually increased with an increase in Na content when Na was added at less than 0.25 wt%. In addition, the temperature for the peak gradually shifted to a low temperature, which becomes more appropriate for bismuth telluride application scenarios. The maximum *ZT* ≈1.03 was achieved at 303 K for the sample with 0.25 wt% Na, which was more than 70% higher than the sample without Na. The samples with Na contents of 0.1 wt% and 0.2 wt%, although not the best performing, demonstrated an overall increase in the whole measured temperature range. Generally, an increase in the *ZT* value caused a significant increase in the average *ZT* value (*ZT_ave_*), which can be obtained by the following formula:

(6) $${\mathrm{ZT}}_{\mathrm{ave}}=\frac{\int_{T_{c}}^{T_{h}}\mathrm{ZTdT}}{T_{h}-T_{c}}$$

where *T_h_* and *T_c_* are the temperatures of the hot side and cold side, respectively, which are 323 and 398 K, respectively. Once a trace amount of Na was doped, the *ZT*_ave_ significantly increased when x ≤ 0.25. A high *ZT*_ave_ greater than 0.9 can be obtained when *x* = 0.2 and 0.25. The increase in *ZT*_ave_ indicates the potential for practical application. A large range of Na contents means higher fault tolerance in industrial production. Thus, adding Na is confirmed to be a promising strategy for enhancing the TE performance of n‐type polycrystal Bi_2_Te_3_ materials.

**Figure 6:**
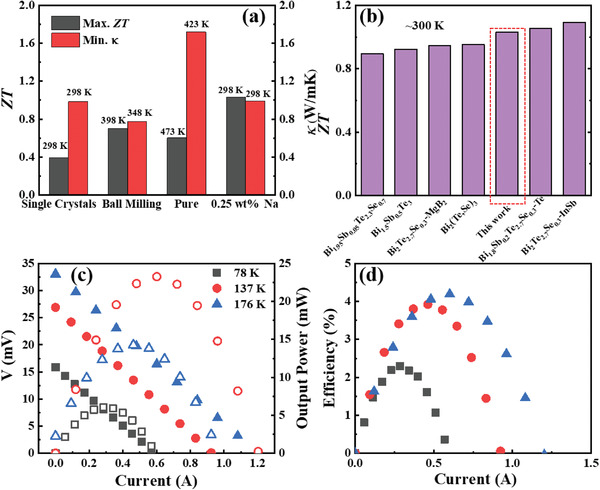
a) Maximum *ZT* value and minimum thermal conductivity with the corresponding temperatures of the sample with 0.25 wt% Na in this work and pure Bi_2_Te_3_ prepared by different means. b) Thermoelectric figure of merit comparison of Bi_2_Te_3_‐based materials near 300 K, the data were taken from.^[^ ^41^, ^42^, ^43^, ^44^, ^45^, ^46^ ^]^ The electric current dependence of the c) output voltage, output power, and d) TE energy conversion efficiency for the sample with 0.25 wt% Na.

**Figure** **6** displays the maximum *ZT* value and minimum *κ* with the corresponding temperatures of the sample with 0.25 wt% Na in this work and pure Bi_2_Te_3_ prepared by different means. Doping with 0.25 wt% Na decreased the minimum *κ* to the level of single crystals and was even similar to that of the sample prepared by ball milling. The maximum *ZT* value for the sample with 0.25 wt% Na was much higher than that of pure Bi_2_Te_3_, regardless of how it was prepared. The corresponding temperatures for the maximum *ZT* value and minimum *κ* for the sample with 0.25 wt% Na were also maintained at 298 K as single crystals, which ensured a potential high efficiency at near temperature. Besides, the peak ZT near 300K for Bi_2_Te_3_‐0.25 wt% Na specimen is comparable to that of other Bi_2_Te_3_‐based composites as shown in Figure 6b. Figure 6c,d exhibit the output voltage, output power, and efficiency increased with an increase in temperature differences for a single leg sample with copper electrode and Ni transition layer. An appreciable efficiency of 4.2% was obtained with a temperature difference of only 176 K. The temperature difference of 78 K can also cause a high efficiency of 2.3%. The high efficiency obtained at such a low‐temperature difference reflected the high TE performance in the low‐temperature range, which is comparable to other Bi_2_Te_3_‐based TE modules in the literature.^[^ ^45^, ^47^ ^]^

**Figure 7:**
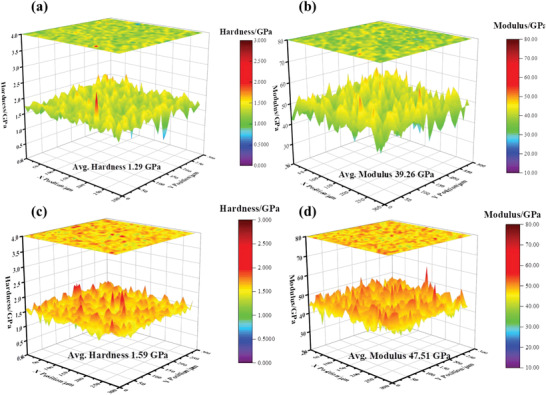
3D contour map of the a) hardness and b) Young's modulus for the pure Bi_2_Te_3_ sample and c,d) for the Bi_2_Te_3_ sample with 0.25 wt% Na.

The 3D contour map of mechanical properties for the pure Bi_2_Te_3_ sample and *x* = 0.25 sample were investigated by the nanoindentation method (nanoindentation force was 5 mN), as demonstrated in **Figure** **7**. The hardness and Young's modulus showed large differences in the variety of positions for both samples, displaying mountain‐like fluctuations in the map. A possible reason for the position dependence of mechanical properties might be the diverse orientation of the laminar grains. More notably, the hardness and Young's modulus of the sample with 0.25 wt% Na content were generally higher than those of the pure Bi_2_Te_3_ sample. The average hardness and Young's modulus of the pure Bi_2_Te_3_ sample were 1.29 and 39.26 GPa, respectively; after doping with 0.25 wt% Na, these values increased to 1.59 and 47.51 GPa, respectively. The hardness and Young's modulus are positively correlated with the strength of chemical bonds. However, the reduced *v*_T_ in Table 1 reflected that the strength of chemical bonds was reduced after doping Na, indicating that there should be another reason for the enhanced mechanical properties.^[^ ^48^, ^49^ ^]^ It is clear to observe that the density of all samples shows an increment with adding Na (Figure S3e, Supporting Information). The high carrier concentration of pure sample shows that the defects of V_Te_ were generated widely in matrix, this kind of evaporation could introduce pores into grains to decrease density. By introducing Na, the escaped Te in melting or sintering process could be trapped in bulk sample and reacted with extra Na element forming the compound Na_2_TeO_4_ which could fill some of the pores located at grain boundaries. Compared with the pores and normal grain boundaries, the increased relative density and complex semi‐coherent interfaces always increase the strength of bulk materials. Finally, the IFFT images (Figure 2c,d) show that there are lots of dislocation cores inside of the grains, which also introduce obvious lattice distortion and strain to increase the ability of dislocation strengthening.

## 3 Conclusions

In summary, the TE performance of n‐type Bi_2_Te_3_ was significantly improved via Na doping. The defects modulated by this strategy synergistically optimized the electrical and thermal transport properties. Specifically, the properly moved Fermi level significantly improved the Seebeck coefficient. The correspondingly decreased carrier concentration not only weakened the interactional scattering between two charge carriers but also decreased the electronic thermal conductivity. The phonon scattering was overall enhanced in the whole temperature range, which may be attributed to the synergistic scattering effect via the point defects and the potentially introduced dislocations. A maximum *ZT* value of 1.03 at 303 K and a *ZT*_ave_ of >0.9 for the range of 323–398 K were achieved with a Na content of 0.25 wt%. This improved peak *ZT* value greater than 70% compared with undoped n‐type Bi_2_Te_3_ reveals the great potential of Na for n‐type Bi_2_Te_3_. The high TE energy conversion efficiency of 4.2% for a low‐temperature difference of 176 K and the enhanced mechanical properties revealed the promising application perspective for Na doping.

## 4 Experimental Section

### Sample Synthesis

The sample was synthesized by the melting method combined with spark plasma sintering. The experimental details were shown in the supporting information.

### Characterization

The phase composition of the materials was analyzed by X‐ray diffraction. The Seebeck coefficient and electrical conductivity were measured by ZEM‐3. The thermal diffusivity *D* was measured by LFA 457. The microstructure of the samples was observed by scanning electron microscopy and transmission electron microscopy. The output power and efficiency of a single leg sample were measured by a thermoelectric conversion efficiency tester. The Vickers hardness and Young's modulus of the sample were measured by the nanoindentation method. The experimental details are shown in the supporting information.

## Conflict of Interest

The authors declare no conflict of interest.

## Supporting information

Supporting Information

## Data Availability

The data that support the findings of this study are available from the corresponding author upon reasonable request.

## References

[1] C. Forman , I. K. Muritala , R. Pardemann , B. Meyer , Renewable Sustainable Energy Rev. 2016, 57, 1568.

[2] F. J. DiSalvo , Science 1999, 285, 703. 10426986 10.1126/science.285.5428.703

[3] G. J. Snyder , E. S. Toberer , Nat. Mater. 2008, 7, 101. 10.1038/nmat2090 18219332

[4] X. ‐ L. Shi , J. Zou , Z. ‐ G. Chen , Chem. Rev. 2020, 120, 7399. 32614171 10.1021/acs.chemrev.0c00026

[5] J. ‐ F. Li , W. ‐ S. Liu , L. ‐ D. Zhao , M. Zhou , NPG Asia Mater 2010, 2, 152.

[6] J. He , T. M. Tritt , Science 2017, 357, 6358. 10.1126/science.aak9997 28963228

[7] C. Li , Yi Wu , Yi‐X Zhang , J. Guo , J. Feng , Z. ‐. H. Ge , Mater. Lab 2022, 1, 220014.

[8] J. Pei , B. Cai , H. ‐ L. Zhuang , J. ‐ F. Li , Natl. Sci. Rev. 2020, 7, 1856. 34691527 10.1093/nsr/nwaa259 PMC8290941

[9] T. J. Zhu , L. P. Hu , X. B. Zhao , J. He , Adv. Sci. 2016, 3, 1600004. 10.1002/advs.201600004 PMC5071658 27818905

[10] N. Dragoe , Mater. Lab 2022, 1, 220001.

[11] B. C. Qin , L. ‐ D. Zhao , Mater. Lab 2022, 1, 220004.

[12] J. Li , Q. Tan , J. F. Li , D. W. Liu , F. Li , Z. Y. Li , M. Zou , K. Wang , Adv. Funct. Mater. 2013, 23, 4317.

[13] Q. Hu , W. Qiu , L. Chen , J. Chen , L. Yang , J. Tang , ACS Appl. Mater. Interfaces 2021, 13, 38526. 34346229 10.1021/acsami.1c12722

[14] K. Kim , G. Kim , H. Lee , K. H. Lee , W. Lee , Scr. Mater. 2018, 145, 41.

[15] H. ‐ S. Kim , N. A. Heinz , Z. M. Gibbs , Y. Tang , S. D. Kang , G. J. Snyder , Mater. Today. 2017, 20, 452.

[16] T. Fang , X. Li , C. Hu , Q. Zhang , J. Yang , W. Zhang , X. Zhao , D. J. Singh , T. Zhu , Adv. Funct. Mater. 2019, 1900677, 1900677.

[17] L. D. Zhao , B. ‐ P. Zhang , J. ‐ F. Li , H. L. Zhang , W. S. Liu , Solid State Sci. 2008, 10, 651.

[18] Y. Pan , J. ‐ F. Li , NPG Asia Mater 2016, 8, e275. 10.1038/am.2016.9 PMC5091659 27818718

[19] L. Hu , Y. Zhang , H. Wu , Y. Liu , J. Li , J. He , W. Ao , F. Liu , S. J. Pennycook , X. Zeng , Adv. Funct. Mater. 2018, 28, 1803617.

[20] S. Il Kim , K. H. Lee , H. A. Mun , H. S. Kim , S. W. Hwang , J. W. Roh , D. J. Yang , W. H. Shin , X. S. Li , Y. H. Lee , G. J. Snyder , S. W. Kim , Science 2015, 348, 109. 25838382 10.1126/science.aaa4166

[21] L. Hu , T. Zhu , X. Liu , X. Zhao , Adv. Funct. Mater. 2014, 24, 5211.

[22] H. ‐ L. Zhuang , J. Pei , B. Cai , J. Dong , H. Hu , F. Sun , Y. Pan , G. J. Snyder , J. Li , Adv. Funct. Mater. 2021, 31, 2009681.

[23] L. D. Zhao , B. ‐ P. Zhang , J. ‐ F. Li , M. Zhou , W. S. Liu , Phys. B: Condens. Matter. 2007, 400, 11.

[24] L. P. Hu , X. H. Liu , H. H. Xie , J. J. Shen , T. J. Zhu , X. B. Zhao , Acta Mater. 2012, 60, 4431.

[25] J. Navrátil , Z. Starý , T. Plechác̆ek , Mater. Res. Bull. 1996, 31, 1559.

[26] Y. Pan , T. R. Wei , C. F. Wu , J. F. Li , J. Mater. Chem. C 2015, 3, 10583.

[27] W. G. Zeier , A. Zevalkink , Z. M. Gibbs , G. Hautier , M. G. Kanatzidis , G. J. Snyder , Angew. Chem., Int. Ed. 2016, 55, 6826. 10.1002/anie.201508381 27111867

[28] W. ‐ S. Liu , Q. Zhang , Y. Lan , S. Chen , X. Yan , Q. Zhang , H. Wang , D. Wang , G. Chen , Z. Ren , Adv. Energy Mater. 2011, 1, 577.

[29] Y. Pan , U. Aydemir , F. H. Sun , C. F. Wu , T. C. Chasapis , G. J. Snyder , J. F. Li , Adv. Sci. 2017, 4, 1700259. 10.1002/advs.201700259 PMC5700642 29201622

[30] R. Kumar , R. Bhatt , A. Tewary , A. K. Debnath , P. Bhatt , N. Mani , P. Jha , P. Patro , S. Bhattacharya , M. Pathak , M. K. Khan , A. Singh , K. P. Muthe , J. Mater. Chem. C 2022, 20, 7970.

[31] Y. K. Zhu , Y. Sun , J. Zhu , K. Song , Z. Liu , M. Liu , M. Guo , X. Dong , F. Guo , X. Tan , B. Yu , W. Cai , J. Jiang , J. Sui , Small 2022, 18, 2201352. 10.1002/smll.202201352 35429134

[32] A. Hashibon , C. Elsässer , Phys. Rev. B 2011, 84, 144117.

[33] G. R. Miller , C. ‐ Y. Li , J. Phys. Chem. Solids 1965, 26, 173.

[34] M. Hong , W. Lyu , Y. Wang , J. Zou , Z. G. Chen , J. Am. Chem. Soc. 2020, 142, 2672. 31940193 10.1021/jacs.9b13272

[35] Z. M. Gibbs , H. ‐ S. Kim , H. Wang , G. J. Snyder , Appl. Phys. Lett. 2015, 106, 022112.

[36] M. Hong , Z. G. Chen , J. Zou , Chinese Phys. B 2018, 27, 048403.

[37] P. H. M. Böttger , G. S. Pomrehn , G. J. Snyder , T. G. Finstad , Phys. Status Solidi Appl. Mater. Sci. 2011, 208, 2753.

[38] P. G. Klemens , Proc. Phys. Soc., Sect. A. 1955, 68, 1113.

[39] Y. Pan , U. Aydemir , J. A. Grovogui , I. T. Witting , R. Hanus , Y. Xu , J. Wu , C. ‐ F. Wu , F. ‐ H. Sun , H. ‐ L. Zhuang , J. ‐ F. Dong , J. ‐ F. Li , V. P. Dravid , G. J. Snyder , Adv. Mater. 2018, 30, 1802016. 10.1002/adma.201802016 29984538

[40] Y. Sun , H. Qin , W. Wang , F. Guo , W. Cai , J. Sui , ACS Appl. Energy Mater. 2021, 4, 4986.

[41] L. Hu , Y. Zhang , H. Wu , Y. Liu , J. Li , J. He , W. Ao , F. Liu , S. J. Pennycook , X. Zeng , Adv. Funct. Mater. 2018, 28, 1803617.

[42] Y. Zhou , F. Meng , J. He , A. Benton , L. Hu , F. Liu , J. Li , C. Zhang , W. Ao , H. Xie , ACS Appl. Mater. Interfaces 2020, 12, 31619. 32539321 10.1021/acsami.0c07566

[43] B. Chen , J. Li , M. Wu , L. Hu , F. Liu , W. Ao , Y. Li , H. Xie , C. Zhang , ACS Appl. Mater. Interfaces 2019, 11, 45746. 31729854 10.1021/acsami.9b16781

[44] Y. Wu , Y. Yu , Q. Zhang , T. Zhu , R. Zhai , X. Zhao , Adv. Sci. 2019, 6, 1901702. 10.1002/advs.201901702 PMC6839625 31728293

[45] B. Zhu , Xi. Liu , Q. Wang , Y. Qiu , Z. Shu , Z. Guo , Y. Tong , J. Cui , M. Gu , J. He , Energy Environ. Sci. 2020, 7, 2106.

[46] D. Li , J. M. Li , J. C. Li , Y. S. Wang , J. Zhang , X. Y. Qin , Y. Cao , Y. S. Li , G. D. Tang , J. Mater. Chem. A 2018, 6, 9642.

[47] R. Deng , X. Su , S. Hao , Z. Zheng , M. Zhang , H. Xie , W. Liu , Y. Yan , C. Wolverton , C. Uher , M. G. Kanatzidis , X. F. Tang , Energy Environ. Sci. 2018, 11, 1520.

[48] Y. ‐ K. Zhu , J. Guo , Y. ‐ X. Zhang , J. ‐ F. Cai , L. Chen , H. Liang , S. ‐ W. Gu , J. Feng , Z. ‐ H. Ge , Acta Mater. 2021, 218, 117230.

[49] G. Zheng , X. Su , T. Liang , Q. Lu , Y. Yan , C. Uher , X. Tang , J. Mater. Chem. A 2015, 3, 6603.

