# Supplementary material for: Tunable Electrical Conductivity and Simultaneously Enhanced Thermoelectric and Mechanical Properties in n‐type Bi2Te3
Source: Adv Sci (Weinh). 2022 Jul 28;9(27):2203250. doi: 10.1002/advs.202203250 (PMC9507343; doi:10.1002/advs.202203250)
Supplement: Supplementary file 1 — Supporting Information [file ADVS-9-2203250-s001.pdf]

ADVANCED SCIENCE
Open Access

Supporting Information

for *Adv. Sci.*, DOI 10.1002/advs.202203250

Tunable Electrical Conductivity and Simultaneously Enhanced Thermoelectric and Mechanical Properties in n-type Bi_2Te_3

Lu-Yao Lou, Jianmin Yang, Yu-Ke Zhu, Hao Liang, Yi-Xin Zhang, Jing Feng, Jiaqing He, Zhen-Hua Ge and Li-Dong Zhao**

Supplementary Information

Tunable Electrical Conductivity and Simultaneously Enhanced Thermoelectric and Mechanical Properties in n-type Bi_2Te_3

Lu-Yao Lou[1], Jianmin Yang[2], Yu-Ke Zhu[1], Hao Liang[1], Jing Feng[1], Jiaqing He[2], Zhen-Hua Ge[1] and Li-Dong Zhao[3*]*

1. Faculty of Materials Science and Engineering, Kunming University of Science and Technology, Kunming 650093, China.
2. Shenzhen Key Laboratory of Thermoelectric Materials and Department of Physics, Southern University of Science and Technology, Shenzhen 518055, China
3. School of Materials Science and Engineering, Beihang University, Beijing 100191, China

*Corresponding author: Z.-H. Ge (zge@kust.edu.cn), L.-D. Zhao (zhaolidong@buaa.edu.cn)

I. Experimental details

Sample Synthesis

The raw elements Bi (powder, 99.99 % Aladdin), Te (particle, 99.99 % Macklin), and Na (block, 99.9 %) in stoichiometric proportions of Bi_2Te_3+*x* wt.% (*x* = 0.1, 0.2, 0.25, and 0.3) were weighed out in a glove box and encapsulated in quartz tubes with 10^{-4} Pa. Then the tubes were put into the furnace and melted at 1223 K for 10 hours. The as-prepared ingots were broken by a high-energy ball milling machine (Retsch Emax, Germany) for 30 min at 800 rpm under a protective atmosphere (95 vol.% Ar, 5 vol.% H_2). Then, the ball-milled powders were poured into a ϕ15 mm graphite die and sintered via a spark plasma sintering (SPS) method (Dr. Sintering, lx-632, Japan) under 50 MPa and 723 K in vacuum (< 4 Pa) for 5min. In accordance with the test requirements below, the obtained bulk samples were cut and polished.

Characterization

The Archimedes method was adopted to measure the density ρ. The phase composition of the materials was analyzed by X-ray diffraction test (MiniFlex600, Rigaku, Japan) with Cu Kα radiation (λ = 1.5406 Å). The Seebeck coefficient/electrical resistivity measurement system (ZEM-3-M8, Ulvac-Riko, Japan) was used to measure the Seebeck coefficient and electrical conductivity. The laser flash method (LFA457, Netzsch, Germany) was used to measure thermal diffusivity D. The thermal conductivity was obtained by the product of $DC_p\rho$, where C_p is the specific heat calculated with the Dulong-Petit limit. The electronic thermal conductivity (κ_e) was calculated according to Wiedeman-Franz's Law: $\kappa_e = \sigma LT$, where the Lorenz factor (L) was roughly calculated by the equation[1]: $L = 1.5 + \exp(-\frac{|S|}{116})$. Hall coefficient tester (HMS-7000, Ecopia, Korea) was used to test the carrier concentration and carrier mobility. The pictures of the microstructure for all bulk samples were taken by scanning electron microscopy (ZEISS, Sigma 300, Germany). A thermoelectric conversion efficiency tester (Mini-PEM, Ulvac-Riko, Japan) was used to measure the output power and efficiency of each leg. The Nanoindentation method (iMicro, KLA, USA) was used to measure mechanical properties, Vickers hardness, and Young's modulus.

II. EDS mapping of Bi_2Te_3+0.25 wt% Na sample

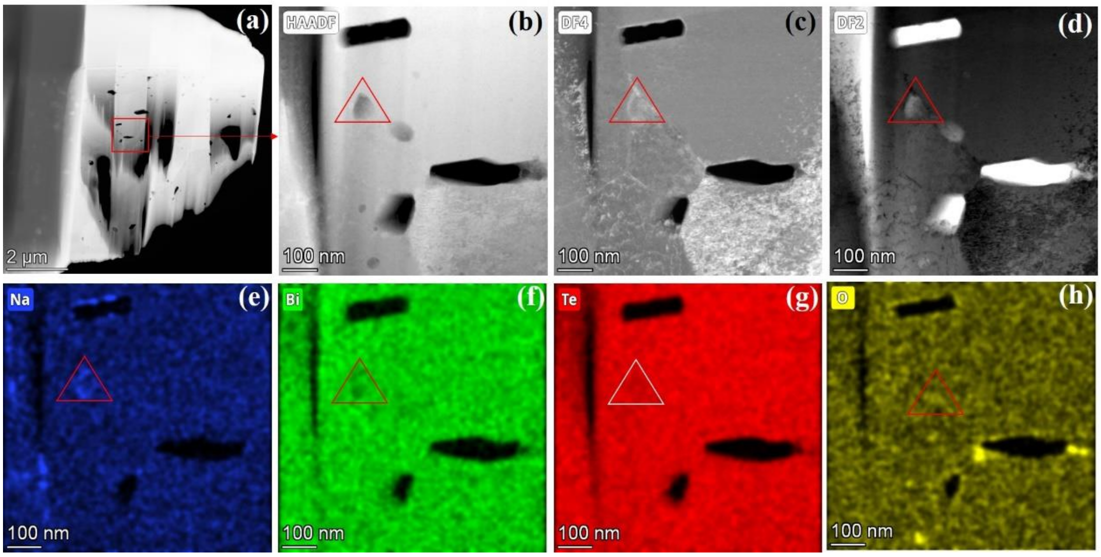

Fig. S1 (a) Overview HAADF-STEM image of Bi_2Te_3+0.25 wt% Na sample.（b-d）Low magnification STEM image for Bi_2Te_3+0.25 wt% Na sample. corresponding to the red rectangle marked in (a). (d)-(g) EDS-STEM elemental mapping of Na, Bi, Te

and O corresponding to (b). Showing that elements Na, Te and O would segregation in the red region as shown in figure (a).

III. FESEM images of Na doped Bi_2Te_3 bulk sample

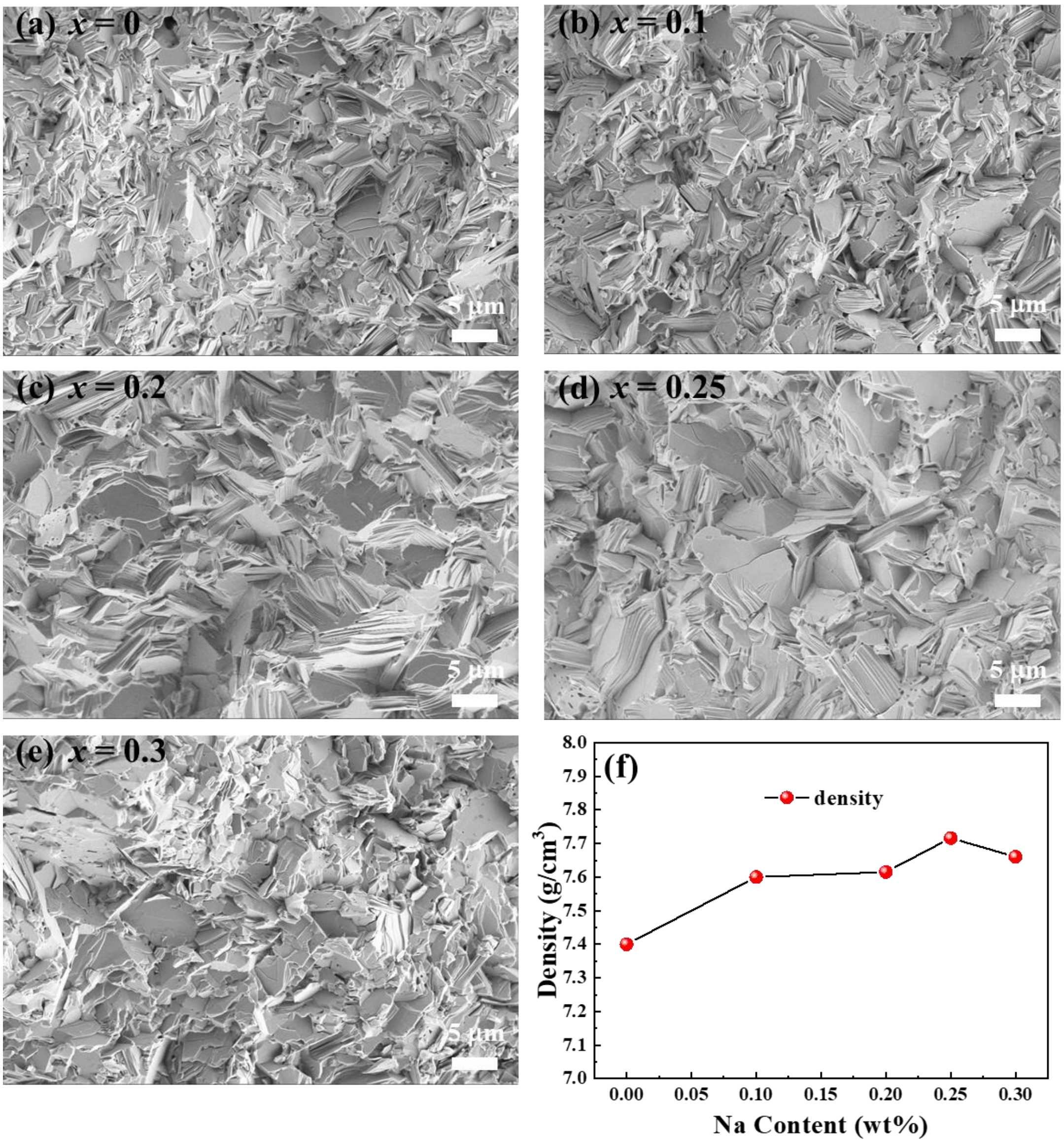

Fig. S2 (a)-(e) SEM images of the fracture surfaces for the Bi_2Te_3+*x* wt.% Na samples. (f) the density of Bi_2Te_3 with different Na content.

IV. additional the electrical conductivity, Seebeck coefficient, and power factor of Bi_2Te_3 +*x* wt% (x=0, 0.3, 0.5) Na bulk samples.

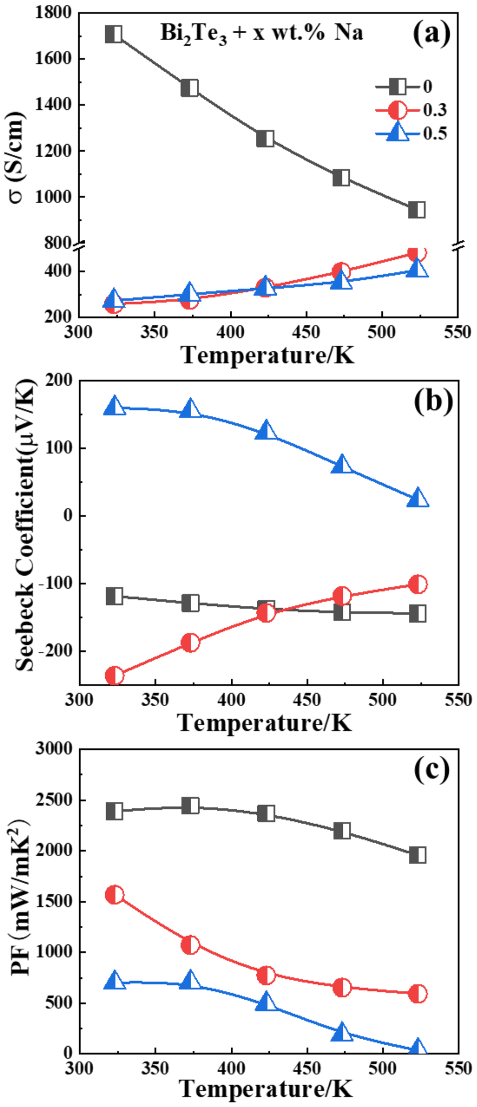

Fig. S3 The temperature dependence of (a) electrical conductivity, (b) Seebeck coefficient, (c) power factor for Bi_2Te_3 with 0, 0.3, 0.5 Na content

References

[1]H.-S. Kim, Z. M. Gibbs, Y. Tang, H. Wang, G. J. Snyder, Characterization of Lorenz number with Seebeck coefficient measurement, APL Mater. 3 (2015) 041506. https://doi.org/10.1063/1.4908244.
